# Dectin-1 plays a redundant role in the immunomodulatory activities of β-glucan-rich ligands *in vivo*

**DOI:** 10.1016/j.micinf.2013.03.002

**Published:** 2013-06

**Authors:** Mohlopheni J. Marakalala, David L. Williams, Jennifer C. Hoving, Rolf Engstad, Mihai G. Netea, Gordon D. Brown

**Affiliations:** a Division of Immunology, Institute of Infectious Diseases and Molecular Medicine, University of Cape Town, South Africa; b Department of Surgery, James H. Quillen College of Medicine, East Tennessee State University, USA; c Biotec BetaGlucans AS, Sykehusveien 23, 9294 Tromsø, Norway; d Department of Medicine, Radboud University Nijmegen Medical Center, The Netherlands; e Section of Immunology and Infection, Institute of Medical Sciences, University of Aberdeen, UK

**Keywords:** Innate immunity, Immunomodulation, Dectin-1, Beta-glucan, MODS, *Staphylococcus aureus*

## Abstract

β-Glucans are known for their ability to trigger both protective and damaging immune responses. Here we have explored the role of the beta-glucan receptor Dectin-1 in archetypical models of protective and non-protective immunomodulation induced by beta-glucan rich ligands. In the first model, we explored the role of Dectin-1 in the ability of soluble purified β-glucans to mediate protection against systemic *Staphylococcus aureus* infection in mice. In the second model, we explored the role of Dectin-1 in zymosan induced multiple organ dysfunction syndrome. In both cases, these β-glucan rich compounds had marked effects *in vivo* which were unaltered by Dectin-1 deficiency, suggesting that this receptor has a redundant role in these murine models.

## 1 Introduction

β-Glucans are glucose polymers that are found in fungal cell walls, plants and some bacteria [1]. β-Glucans are known for their ability to activate leukocytes, and have been of considerable interest as immune modulators, promoting anti-tumorigenic and anti-microbial activities [2]. Administration of these carbohydrates to mice, for example, has been shown to protect against infection with *Staphylococcus aureus* [3], a gram positive opportunistic pathogen that causes soft tissue infections that can lead to invasive disease and sepsis. However, exaggerated inflammatory response that can be induced by β-glucans in certain circumstances can also have detrimental effects, especially if these carbohydrates are administered in particulate form. One relevant example is the ability of a high dose of the β-glucan rich particle zymosan to induce multiple organ dysfunction syndrome (MODS) in mice, which is characterized by uncontrolled systemic inflammation leading to a deterioration of function in several organs [4]. How β-glucans actually mediate these effects on immune function is still unclear.

Several receptors for β-glucans have been identified, including Dectin-1, a receptor we have shown to be expressed by various immune cells including, dendritic cells, monocytes, macrophages, neutrophils and a subset of T cells [5]. This pattern recognition receptor contains a single extracellular lectin-like carbohydrate recognition domain and a cytoplasmic tail with an immunoreceptor tyrosine-based activation-like motif (ITAM-like), which can initiate intracellular signalling upon engagement of β-glucans [5]. The recognition of β-glucans by Dectin-1 induces numerous cellular responses, including phagocytosis, the respiratory burst, the production of arachidonic acid metabolites, and the induction of a number of cytokines and chemokines [5]. We and others have shown that through the recognition of β-glucans, Dectin-1 plays a key role in anti-fungal immunity in both mouse and humans [6]. In this study, we have investigated the involvement of Dectin-1 in both protective and non-protective models of immunomodulation induced by β-glucan rich ligands.

## 2 Materials and methods

### 2.1 Mice

Age-matched (8–10 week-old) male wild-type and *Clec7a*^−/−^ mice on 129/Sv [7] or C57BL/6 background (mice were backcrossed for at least nine generations) were used in these experiments, as indicated. All mice were obtained from the specific-pathogen-free facility of the University of Cape Town. All animal experiments were repeated at least once, and were performed according to animal care and welfare protocols approved by the University of Cape Town Animal Research ethics committee. All experiments utilized a minimum of six mice per group.

### 2.2 *Staphylococcus aureus* infection model

*S. aureus* (ATCC 25923) was obtained from the Medical Microbiology Laboratory of the University of Cape Town. Bacteria were cultured for 6 h in LB medium, washed in PBS and frozen in aliquots at −80 °C. These aliquots were subsequently used for infection into animals. Colony forming units (CFU) were determined by serial dilution onto LB-agar plates.

For infections, mice were infected systemically with 5 × 10^6^ CFU of the bacteria (day 0). In some animals, β-glucans were administered i.v. on day-7 and day-4 with 200 μl containing 1 mg of soluble β-glucan diluted in PBS. Clinical grade highly purified soluble β-glucans were kindly provided by Biotec Pharmacon, Norway. Endotoxin levels were below 0.05 EU/ml. Animals were subsequently monitored and sacrificed when they became moribund or had greater than 20% weight loss. Differences in the gross pathologies of the kidneys from infected mice at day 11 were determined by staining 10% formaldehyde-fixed organ sections with Haematoxylin and Eosin (H&E) stains.

### 2.3 MODS model

For these experiments, we used a model of zymosan-induced generalized inflammation, as described [4,8]. In brief, mice were pre-treated with 40 μg lipopolysaccharide (LPS, Sigma), administered intraperitoneally (ip), 6 days before the i.p. administration of 17 mg of the β-glucan-rich fungal cell-wall-derived particle, zymosan A. Zymosan A (Sigma) was prepared by suspension in paraffin (Sigma), disaggregation by sonication for 60 min, followed by heating for 90 min at 100 °C. Animals were monitored for survival, as described above.

### 2.4 Statistics

All numerical data were analysed using GraphPad Prism 4 software. Survival data were analysed with the log rank test. Results were considered statistically significant with *P* values of less than 0.05.

## 3 Results

### 3.1 The β-glucan mediated protection against *S. aureus* infection is Dectin-1 independent

**Fig. 1:**
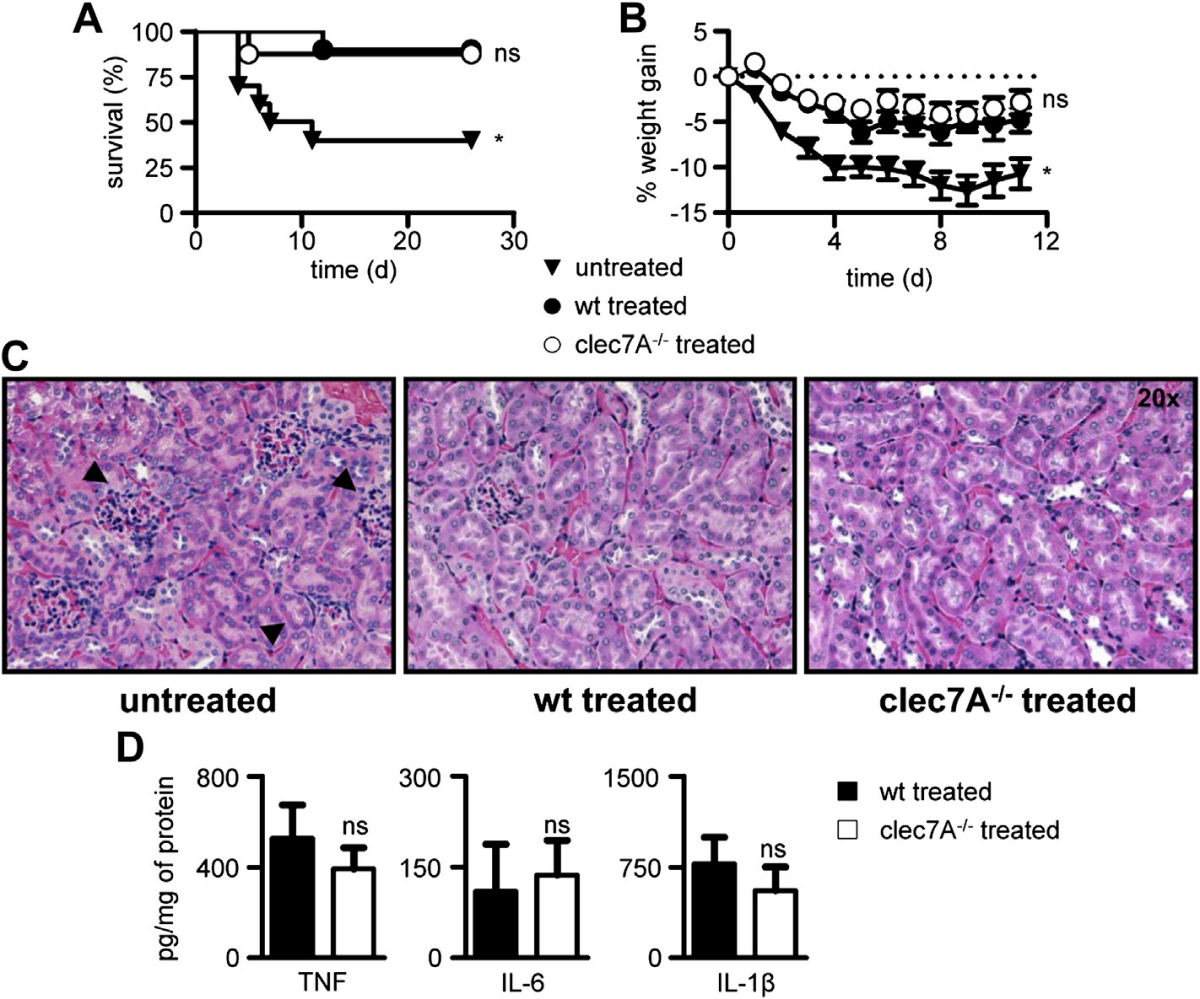
β-Glucan mediated resistance against *S. aureus* infection is independent of Dectin-1. Wild-type (wt) and *clec7A*^−/−^ 129Sv mice were left untreated or treated intravenously with 1 mg of soluble β-glucan at day-7 and -4 prior to infection with 5 × 10^6^ CFU *S. aureus*, as indicated. Animals were monitored daily for mortality (A) and percentage weight gain (B). *, *P* < 0.05. *n* = 8–10 animals per group. (C) H&E histopathology of kidneys from infected animals at day 11, as indicated. (D) Levels of various cytokines in the kidneys of treated and infected mice at day 11, as indicated. *n* = 6 animals per group. ns, not significant.

The administration of soluble β-glucans has been shown to increase resistance to *S. aureus* infection in mice [9,10]. To explore the role of Dectin-1, a major leukocyte receptor for these carbohydrates, we established a systemic model of infection with *S. aureus* which resulted in rapid weight loss and more than 50% mortality in untreated mice (Fig. 1A, B). Moreover, infection of these animals led to marked pathological changes in renal cortex and renal parenchyma, characterized by abscess formation and extensive inflammatory cell infiltration (Fig. 1C) [10]. As previously reported [10], pre-treatment of mice with a highly purified, clinical grade, soluble β-glucan prior to infection with *S. aureus*, significantly increased the ability of wild-type mice to resist the infection (Fig. 1A), which corresponded to reduced weight loss (Fig. 1B) and reduced pathology in the kidneys (Fig. 1C). However, pre-treated mice lacking Dectin-1 showed similarly enhanced survival and reduction in weight loss and kidney pathology (Fig. 1A–C). Moreover, we did not detect any difference between treated wild-type and Dectin-1 deficient mice in the levels of several cytokines known to be influenced by Dectin-1 [5] (Fig. 1D). Thus these results indicate that while pre-treatment of mice with β-glucans can provide protection against systemic *S. aureus* infections, Dectin-1 plays a redundant role in this process.

### 3.2 Dectin-1 is not involved in zymosan-induced multiple organ dysfunction syndrome (MODS)

The administration of a single high dose of zymosan in mice induces the onset of multiple organ dysfunction syndrome (MODS), and is a well-known animal model for studying the underlying mechanisms involved in the development of this disease [4]. Zymosan is a cell wall extract from *Saccharomyces cerevisiae* that is rich in biologically active beta-glucans and we have previously shown that Dectin-1 is the main receptor for unopsonised zymosan on leukocytes, triggering several cellular responses to these particles [11]. To determine whether Dectin-1 was involved in zymosan-induced MODS, we compared the development of the disease in wild-type and Dectin-1-deficient mice.

**Fig. 2:**
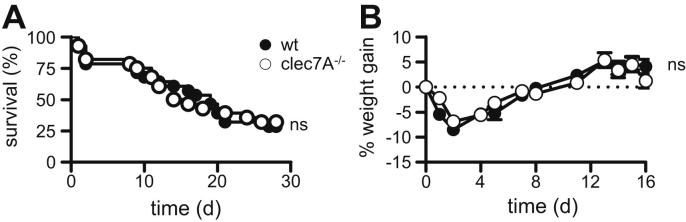
The development of zymosan induced MODS occurs independently of Dectin-1. Survival (A) and percentage weight gain (B) of wild type (wt) and *clec7A*^−/−^ C57BL/6 mice (*n* = 28 animals per group) following the administered of 17 mg of zymosan (day 0). Mice were pre-treated with 40 μg LPS, as described in the materials and methods. Survival data were analysed with the log rank test. ns, not significant.

Following the administration of a single dose of zymosan, both groups of animals showed similar patterns of survival and a typical three-phasic disease known to be associated with this inflammatory model [4]. In the first phase, we observed ∼20% mortality in both groups within the first 2 days after zymosan administration (Fig. 2A); this acute phase of the illness was also characterized by symptoms such as diarrhoea, ruffled fur, lethargic behaviour and weight loss (Fig. 2B and data not shown). Between day 3 and 7 (the second phase), the surviving animals in both groups appeared to return to normal health, as reflected by the display of typical behaviour, smooth fur and normal faeces. No mortality was observed in this phase, and mice started gaining weight (Fig. 2B and data not shown). The third phase which is thought to resemble clinical progression of MODS in humans [4], occurred after day 8, with mortality and a re-emergence of clinical symptoms (Fig. 2 and data not shown). Both the wild-type and Dectin-1 deficient mice presented with further 40–45% mortality from day 8 to day 21. Thus, the absence of a difference in disease progression in the Dectin-1 deficient mice indicates that this receptor plays a redundant role in zymosan induced MODS.

## 4 Discussion

β-Glucans are potent immunomodulators that have been demonstrated to possess both therapeutic benefits and well as detrimental effects when administered *in vivo*, depending on the disease and experimental model [1,2]. The ability of these carbohydrates to drive these responses is thought to stem, at least in part, from their capacity to trigger the activation of leukocytes [2]. In line with this, recent studies have shown that β-glucans mediate epigenetic reprogramming of circulating monocytes, thus inducing partial protection to reinfection in a T/B-cell independent manner, in a process that has been called *trained immunity* [12]. While several receptors for these carbohydrates have been identified [13], Dectin-1 has been shown to be the major receptor for β-glucans on leukocytes and is capable of mediating the biological activities of these carbohydrates *in vitro* [11,14]. Importantly, the ability of Dectin-1 to recognize β-glucans and induce cellular responses is influenced by the structure of these carbohydrates and the cell type expressing this receptor [15–19].

In this study, we have examined the role of Dectin-1 in two archetypical murine models that display either the beneficial or the detrimental effects of β-glucan rich ligands. In the first model, we determined the contribution of Dectin-1 to the protective effect of highly purified soluble β-glucans during systemic infection with *S. aureus*. Previous studies have shown that the administration of these compounds can significantly enhance resistance to infection and the survival of mice following infection with this Gram-positive bacterium [3,9,10]. Consistent with these prior observations, we found that pre-treatment of mice with intravenously administered soluble β-glucans significantly enhanced their ability to resist subsequent systemic infection with *S. aureus* (Fig. 1). However, the ability of β-glucans to protect mice was not diminished by Dectin-1 deficiency, indicating that this receptor was not involved in mediating these protective responses *in vivo*. While we and others have shown that Dectin-1 can recognize soluble β-glucans *in vitro*, recent evidence from the Underhill laboratory suggests that productive intracellular signalling from Dectin-1 can only occur following recognition of large β-glucan complexes and the exclusion of inhibitory phosphatases from the “phagocytic synapse” [15–17]. Moreover, Dectin-1 involvement in a β-glucan-mediated antitumour model was also recently shown to depend on the nature of the β-glucan employed [20]. Thus the redundant role of Dectin-1 in the model tested in our study is likely to reflect the inability of soluble carbohydrates to sufficiently activate this receptor *in vivo*. On the other hand, soluble β-glucans are able to induce protection in a Gram-positive sepsis model, as shown here. How β-glucans mediate their protective effects is still unclear, but may involve some sort of leukocyte priming [2]. Indeed, it has been recently shown that the non-specific protective effects induced by β-glucans *in vitro* involve changes at the level of histone methylation and functional reprogramming of monocytes [12], and whether similar molecular mechanisms are able to mediate the protective effects of soluble β-glucans *in vivo* awaits further clarification.

In the second model, we explored the involvement of Dectin-1 in a widely used model of generalized inflammation, induced by the intraperitoneal administration of unopsonised zymosan, which is thought to reflect MODS in humans [4]. However, despite considerable *in vitro* evidence for a role of Dectin-1 in mediating leukocyte responses to zymosan [7,11], we observed no differences in the onset of MODS when this model was tested in Dectin-1-deficient mice. While the reasons for this are not yet fully understood, recent studies suggest that inflammatory responses induced following the activation of complement by particulate β-glucan rich ligands can compensate in the absence of Dectin-1 [21,22]. Alternatively, zymosan also contains low amounts *Saccharomyces* mannans, and other contaminants, and may induce inflammation in a Dectin-1-independent manner. It is interesting that particulate purified β-glucans themselves are unable to induce this disease, and that LPS is required along with zymosan to induce MODS, suggesting that multiple recognition pathways are involved. In summary, we have addressed the role of Dectin-1 in two models of β-glucan-rich ligand-mediated immunomodulation and conclude that this receptor plays a redundant role in these activities. Other receptors and inflammatory systems known to be involved in recognition of β-glucans should be addressed in future work on these models.
